# Understanding fine dining restaurant dynamics in south Asia: A systematic review

**DOI:** 10.12688/f1000research.161879.1

**Published:** 2025-03-10

**Authors:** Paritosh Dabral, Keith Nigli, Smitha Nayak, Senthilkumaran Piramanayagam, Vijay Shree Dhyani

**Affiliations:** 1 Welcomgroup Graduate School of Hotel Administration (WGSHA), Manipal Academy of Higher Education, Manipal, Karnataka, India; 2 Department of Humanities and Management, Manipal Institute of Technology, Manipal Academy of Higher Education, Manipal, Karnataka, India; 3 Kasturba Medical College, Manipal Academy of Higher Education, Manipal, Karnataka, India

**Keywords:** Fine dining, Service quality, customer satisfaction, restaurant, south Asia, systematic review.

## Abstract

**Background:**

Globalization, urbanization, and economic development has had an influence on the dining out behaviour in general and fine dining in particular. The objective of this systematic review is identifying the factors affecting fine dining selection and to understand the fine dining restaurant dynamics.

**Methods:**

In the context of fine dining, both quantitative and qualitative studies conducted in South Asian countries—Afghanistan, Bangladesh, Bhutan, India, Maldives, Nepal, Pakistan, and Sri Lanka—were included in this review. The review was conducted following Preferred Reporting Items for Systematic Reviews and Meta-Analyses (PRISMA) and summarised using narrative synthesis. The search was conducted on Scopus, Web of Science, MEDLINE (PubMed) and Google Scholar. The two-stage screening was independently done by two authors in two stages (titles/abstracts, and full texts) for inclusion, with discrepancies resolved by involvement of a third reviewer. Data was extracted using a piloted form and the results are presented as a narrative summary and in tabular format.

**Results:**

Fourteen studies met the eligibility criteria after de-duplication and screening and were included in the review. These studies were conducted in Bangladesh, India, Nepal and Pakistan and were mostly quantitative in nature. The outcomes assessed in the studies were factors affecting decision to choose fine dine restaurant, customer satisfaction, customer loyalty, willingness to pay, and food wastage.

**Conclusion:**

There is limited literature on types of evidence available related to fine dining in South Asia. The substantial gap extends to the methodological aspect and the outcomes assessed in the fine dining restaurant segment.

## 1. Introduction

Fine dining is characterized by high-quality food, elegant presentation, attentive and personalized service, and a sophisticated atmosphere ( Lee, 2021). Fine dining establishments are often seen as upscale establishments and are known for providing a luxurious dining experience. The growth of the fine dining sector can be attributed to shifting consumer preferences that have been shaped by large-scale and extensive changes in the social, cultural, and economic milieu ( Kumar et al., 2022; Pingali & Abraham, 2022). To Alsubhi et al. (2023), changes in socio-demographic-economic patterns nudges individuals to spend more on high-quality dining experiences. According to a 2021 report on foodservice industry in Asia, it is estimated that Asians will spend about US$2.4 trillion on food by 2030 ( Richard et al., 2021). The same report further adds that consumer preferences have undergone a noticeable shift in terms of demand for healthier diets, preference for fresh food, safe and traceable sources, sustainable consumption, alternative protein, and online purchasing.

In South Asian countries—and especially in India—where economic liberalisation took place in the last decade of previous century, growth in the previous two decades has led to an increase in disposable income among the middle and upper classes ( Pingali & Khwaja, 2004). This coupled with urbanization, exposure to global cuisine, tourism, usage of social media, changing lifestyles, culinary talent, and government support has led to increase in dining out at establishments that offer local, regional, and global fare ( Kumar et al., 2022). Researchers have hinted at the hedonic nature of fine dining and indicated that customers expect while dining at an upscale restaurant will not only satisfying hunger but also provide hedonic pleasure and opportunities to create memories that can be cherished ( Hlee et al., 2019; Parsa et al., 2020).

While selecting a restaurant, consumers prioritize a variety of factors with food quality and service standing out as primary considerations in attracting patrons to fine dining restaurants ( Chua et al., 2020; Hsu et al., 2018). Nevertheless, customers’ preferences are ever-evolving and subject to numerous factors ( Chua et al., 2020; Khan et al., 2023; Lau et al., 2022): online reviews, restaurant physical settings, menu variety, dining motivation, service digitization, ethical food practices, food safety standards, innovative menu offerings. These attributes play a significant role in influencing restaurant’s attractiveness and can sway customers’ decision. Furthermore, these factors vary according to the restaurant segments ( Guo et al., 2023) and dining occasion ( Chua et al., 2020). Understanding customer preferences in general and the decision-making process in particular have always been an objective of business owners and managers; increasing competition has made it critical. Managers are looking at ways to innovate and make their offerings novel, something that customers find appealing.

A preliminary search on Scopus and Google Scholar was conducted to ascertain that no current scoping or systematic reviews on the topic. The results of this search yielded two related reviews. One literature review, ( Sinha et al., 2019), focused on to the current state of customer satisfaction and factors affecting in fine dining restaurant. However, none of the studies included in this review were from South Asia. The other review ( Medeiros & Salay, 2013) was on food service selection factors in fast food outlets. The authors had included restaurant from all segments and one study was from South Asia. Repeated and varied search queries yielded no further studies that met the inclusion criteria. This indicates that the need for a review was coherent and consistent. While there are several studies that examine the antecedents of restaurant selection ( Ahlawat et al., 2022; Khoa, 2021; Okumus, 2021), the lack of review study in the context of setting (fine dine restaurants) and geographical region (South Asia) highlights the obvious need for a systematic review such as this. The primary objective of this review was to map the literature on scope and breadth of the scientific evidence available on fine dining in South Asia and specifically different aspects and the factors associated with fine dining restaurant selection. Furthermore, the authors believe that this review will serve to indicate gaps and scope for future research.

The authors have been guided by recent research highlighting best practices ( Azarian et al., 2023; Sharma et al., 2023; Xiao & Watson, 2019). The organisation of this review is: In the methods section, list the review questions, set the eligibility criteria, identify information sources, determine search strategy, describe how data were extracted, assessed, and synthesised. The result section explains the characteristics of the studies that were included and describes the results of the systematic review. In the discussion section the authors explain the implications of the results and contextualise the findings. The conclusion section acknowledges the gaps in the current literature and offers other researchers a springboard for future research with reference to aspects such as contexts, methodology, and theory.

## 2. Methods

### 2.1 Review design

The review and the protocol for this review was developed in accordance with Preferred Reporting Items for Systematic reviews and Meta-Analyses (PRISMA) ( Page et al., 2021) and PRISMA – P ( Shamseer et al., 2015) guidelines. This framework is widely used when examining the studies from a methodological, theoretical, and contextual point of view.

### 2.2 Review questions

(A) What is the current evidence on fine dining restaurant dynamics and the gaps in the existing literature on aspects related to fine dining restaurants in South Asia?
(B) What are different aspects and the factors that affect the decision to select fine dining restaurants?

### 2.3 Eligibility criteria

Population included adults (above 18 years of age) and they have been to fine dining restaurants. As authors, we have not followed any specific definition for “fine dining” restaurant. We have included all studies where the customers have been to “fine dine” or “upscale” or “up-market” restaurants where full table service was offered. While all types of study designs—quantitative, qualitative, and mixed methods—were eligible for inclusion, studies that utilize secondary data were excluded from the scope of this study. For this systematic review, reviews, conference papers, letters to the editor, pre-prints, editorials, thesis, commentaries, viewpoints, and perspectives were excluded.

Also, studies conducted in the context of quick service restaurants, fast food outlets, casual dining, family-style, and counter-service restaurants were not included in this review. Lastly, research articles published between January 2011 and October 2024, in English language, and conducted in South Asian countries were eligible for inclusion in this review. According to a regional inter-governmental association, Afghanistan, Bangladesh, Bhutan, India, Maldives, Nepal, Pakistan, and Sri Lanka are considered as South Asian countries ( South Asian Association for Regional Cooperation, 2020).

We have narrowed down the scope of this review to focus exclusively on this region as the regional countries have significant similarities in their cuisine and culture. Furthermore, research conducted in other regions or countries may not accurately reflect the true situation or could potentially exaggerate certain aspects that are not usually endemic to this region.

### 2.4 Information sources and search strategy

Studies were identified by conducting a search on Medline (PubMed), Web of Science, Scopus, and Google Scholar with appropriate filters (language and period). The keywords used for building the search strategy were "fine dining" OR "restaurant dining" OR "upscale restaurant" OR "high-end restaurants" OR "five-star restaurant" OR "full service restaurant” AND “customer satisfaction" OR "customer loyalty" OR "return intention" OR "revisit intention" OR "customer fulfilment" AND “barriers” OR “factors” OR “facilitators” OR "motivating factors" OR "service quality" OR "physical environment" OR "food quality" OR “ambience” OR “menu” OR “pricing”. The search was customized for all other databases. The search strategy is provided in supplementary file 1.

### 2.5 Selection of eligible studies

Rayyan software ( https://www.rayyan.ai/) was used for de-duplication and screening the articles after the database searches ( Ouzzani et al., 2016). After de-duplication, in the first stage, the remaining records were screened by title and abstract and then, in the second stage, full-text articles were screened against the eligibility criteria. In each stage, two reviewers (PD and VSD) independently screened articles. Disagreement between the reviewers was settled by consulting a senior reviewer (SKP and KSN). Search results and two-stage screening process is depicted using PRISMA 2020 flowchart ( Page et al., 2021).

### 2.6 Data extraction and charting

After a detailed discussion between the authors, a data extraction form was designed and pilot-tested to extract data in adequate detail. Modifications proposed by the authors were discussed and then incorporated in the final form. This final version of the form included various categories to capture the data relevant to our review such as data on the study citations, methodological details, fine dining, and the outcomes such as factors affecting fine dining selection, customer satisfaction, return intention, servicescape, customer loyalty. Data extraction was done independently by two reviewers and inconsistencies between the reviewers were discussed with a senior reviewer to reach consensus.

### 2.7 Quality assessment

Qualitative assessment of the included studies was done using Hawker's tool ( Hawker et al., 2002). This tool is widely used in systematic reviews to evaluate studies by scoring them under nine criteria—1 point for each criteria that the study meets—such as title, abstract, introduction, method, sampling etc. The total score for each study ranges from a minimum of 9 to a maximum of 36. Subsequently, the studies, based on the total score, were categorised into three: first category corresponding to 30–36 score, second category for score ranging from 24 to 29, and for third category a score less than 24.

### 2.8 Data synthesis and reporting

The data were summarized narratively with key methodological attributes and results of the included studies presented in tabular format. A descriptive summary of the research methods such as research design, sampling strategy, analysis methods employed and the different aspects of fine dining are presented based on factors affecting customers’ choice, satisfaction, willingness to pay. This systematic review is reported in accordance with PRISMA guidelines ( Page et al., 2021) and is provided in supplementary file 2.

## 3. Results

### 3.1 Search results and study selection

Database search resulted in a total of 1180 records: 291 in Medline (through PubMed), 644 in Scopus, 245 in Web of Science. Review of the results yielded 113 duplicates and they were removed. A total of 1067 records were retained for title and abstract screening. After screening titles and abstracts, 979 articles were excluded. Another 200 citations were searched on Google Scholar. A total of 88 full text articles from database search and 30 from Google Scholar were taken forward for full-text screening. Studies at full text stage were excluded based on following reasons: not conducted in South Asia (n=59), fine dining restaurants not included in the study (n=16), population other than customers of fine dining restaurants (n=10), publication type(n=12), secondary data analysis using online reviews (n=03), and full text unavailable (n = 04). After full-text screening, fourteen studies met the eligibility criteria and were included in this review. Study selection procedure at different stages is depicted in PRISMA flow chart (Extended data).

### 3.2 Characteristics of included studies

The characteristics of the included studies on different aspects of fine dining restaurants are presented in table 1.

**Table 1.** Characteristics of included studies.

Study ID/period	Country	Study setting	Study design	Population	Sample size and sampling method	Research Instrument	Outcome and analysis
Tinne 2012	Dhaka, Bangladesh	Upscale Restaurants	Survey	Customers who have shown interest in survey and have the experience at upscale restaurants	N=75 Non-probability sampling	Questionnaire and Likert Scale	Factors that affect consumer selection criteria about the upscale restaurant Factor analysis
Dutta 2014	New Delhi and the NCR (national capital territory), India	High-end fine dining Casual dining restaurants, Quick-service chain restaurants, and Quick-service independent restaurants	Survey Scenario-based factorial experimental design	Participants who visited a fine dining or a casual dining restaurant in the past two months	N=308 Random sampling	Open-ended questionnaire and Likert scale	Consumer patronage and willingness to pay 2 ^3^ factorial design by analysis of variance (ANOVA)
Sahni 2017 Aug-Oct 2016	Delhi & NCR, India	Fine Dining Restaurant	Survey	Local residents or tourists	N=192 from 10 fine dining restaurants Convenience sampling	Self-administered questionnaire	Factors influencing the selection of fine dining restaurant Chi square
Shahzadi 2017	Islamabad and Rawalpindi, Pakistan	Fine dining restaurants	Not Specified	Customers dining in the fine dining restaurants	N= 296 from 07 fine dining restaurants Non-probability sampling	Self-administered questionnaire and Likert scale	Perceptions of customers regarding the key restaurants attributes Customer satisfaction and behavioral intentions. Correlation and Regression
Shashikala 2018	Bangalore, India	Fine dining restaurant with an average check per-person per meal ranging between Rs. 1000 to Rs. 1500	Survey	Participants who visited a fine dining restaurant	N=422 Two - stage systematic sampling	Self-administered structured questionnaire	Impact of Servicescape Factor analysis
Upreti and Sherpa 2018	Kathmandu, Nepal	Fine dining Restaurants	Survey	Diners of fine dining restaurants	N=197 Random Sampling	Questionnaire and Likert scale	Perceived value Dinning satisfaction Descriptive statistics, correlation, regression analysis, Multicollinearity and F-test
Rai 2019	Jaipur, India	Fine dining Restaurants	Survey	Diners of fine dining restaurants	N= 436	Questionnaire and Likert scale	Behavioral intensions/Customer loyalty Correlation and linear regression model
Gharpure 2021	Nagpur, India	Fine dining Restaurants	Not specified	Diners of fine dining restaurants	N= 178 Convenience sampling	Self-administered questionnaire	Customer satisfaction and Customer’s Post-Dining Behavioral Intentions Descriptive statistics, correlation, ANOVA and t-test
Biswas and Verma 2022 Oct 2019 to Jan 2020	New Delhi and Bengaluru, India	Top 10 restaurants according to Conde Nast Traveler Magazine List, 2019	Survey	Customers of top 10 restaurants	N=520 Purposive sampling	Questionnaire	Customer satisfaction Structural equation modeling and factor analysis
Tahir 2022 Sep- Nov 2020	Islamabad, Lahore, and Faisalabad, Pakistan	Fine dining and casual dining Restaurants	Survey	Diners of fine and casual dining restaurants	N=502 Stratified random sampling	Questionnaire and Likert scale	Diner’s sustainable behavior and wastage reduction practices Structural equational model
Nath 2022	Kolkata, India	Fine dining Casual dining Quick-service restaurants, and enterprise setting restaurants	Survey	Consumers	N=105 Snowball sampling	Questionnaire	Impact of Servicescape on Consumer Perceptions and satisfaction Frequency and Percentage
Gupta 2022 December 2019 to March 2020	Delhi, India	Fine dining Restaurants Trip Advisor’s best luxury fine dining restaurants rankings	Survey	Consumers of fine dining restaurants	N=369 Convenience sampling	Questionnaire	Willingness to pay Structural equation modeling
Lim 2022 2 months in 2020	Chandigarh and New Delhi, India	Fine dining restaurants	Survey	Customers of fine-dining restaurants	N = 693 Purposive sampling	Questionnaire	Customer satisfaction Exploratory and confirmatory factor analysis Structural equation modeling
Zeba 2022	Hyderabad, India.	Fine dining restaurants based on Yellow Pages and travel websites such as TripAdvisor	Survey	Customers of fine-dining restaurants	N=216 Convenience sampling	Questionnaire	Customer delight, and Customer satisfaction Customer loyalty Correlation and regression analysis

**3.2.1 Location**

Out of the fourteen studies, one was from Bangladesh ( Tinne, 2012), ten studies were from India ( Biswas & Verma, 2023; Dutta et al., 2014; Gharpure et al., 2021; Gupta et al., 2022; Lim et al., 2022; Nath & Agarwal, 2022; Rai & Anirvinna, 2019; Sahni & Mohsin, 2017; Shashikala & Suresh, 2018; Zeba et al., 2022), two studies were from Pakistan ( Shahzadi et al., 2018; Tahir et al., 2022) and one study from Nepal ( Upreti & Sherpa, 2018).

**3.2.2 Study setting**

A total of eleven studies were conducted in the context of upscale of fine dining restaurants ( Biswas & Verma, 2023; Gharpure et al., 2021; Gupta et al., 2022; Lim et al., 2022; Rai & Anirvinna, 2019; Sahni & Mohsin, 2017; Shahzadi et al., 2018; Shashikala & Suresh, 2018; Tinne, 2012; Upreti & Sherpa, 2018; Zeba et al., 2022). Three studies considered multiple sectors such as fine dining, casual-dining, quick-service, independent, and chain restaurants ( Dutta et al., 2014; Nath & Agarwal, 2022; Tahir et al., 2022). In three studies, the selection of restaurants was done on the basis of average price of a meal for two—between INR 1000 to 1500—( Shashikala & Suresh, 2018), Conde Nast Traveler Magazine List of 2019 ( Biswas & Verma, 2023), and Tripadvisor rankings ( Gupta et al., 2022). Other studies did not contain details about this aspect. The study by Nath & Agarwal, (2022) examined restaurants that were located within another building (such as malls). As regards the period of data collection, three studies specified the period during which data were collected ( Lim et al., 2022; Sahni & Mohsin, 2017; Tahir et al., 2022).

**3.2.3 Study design**

Majority of the studies ( Biswas & Verma, 2023; Gupta et al., 2022; Nath & Agarwal, 2022; Rai & Anirvinna, 2019; Sahni & Mohsin, 2017; Shashikala & Suresh, 2018; Tahir et al., 2022; Tinne, 2012; Upreti & Sherpa, 2018) used survey method. However, one study employed both survey and scenario-based factorial experimental design ( Dutta et al., 2014) and two studies did not specify any study design ( Gharpure et al., 2021; Shahzadi et al., 2018).

**3.2.4 Population**

Most of the studies described the study population was indicated as individuals those who have visited any upscale or fine dining restaurant ( Biswas & Verma, 2023; Gharpure et al., 2021; Gupta et al., 2022; Lim et al., 2022; Rai & Anirvinna, 2019; Shahzadi et al., 2018; Shashikala & Suresh, 2018; Tinne, 2012; Upreti & Sherpa, 2018; Zeba et al., 2022). While Dutta et al. (2014) and Tahir et al. (2022) surveyed individuals who had been to either a fine dine or a casual restaurant, Nath & Agarwal (2022), surveyed individuals who have patronised any restaurant—casual, quick service or fine dining. Local residents or tourists were participants in one study ( Sahni & Mohsin, 2017). Informed consent to participate was explicitly obtained for one study ( Tinne, 2012). Only in one study, discount coupons were offered as a reward to respondents of the survey ( Zeba et al., 2022).

**3.2.5 Sampling**

Non-probability sampling was employed by two studies ( Shahzadi et al., 2018; Tinne, 2012) where the sampling method was judgemental i.e., based on the researcher’s personal judgement. Convenience/purposive sampling was used in seven studies ( Biswas & Verma, 2023; Gharpure et al., 2021; Gupta et al., 2022; Lim et al., 2022; Rai & Anirvinna, 2019; Sahni & Mohsin, 2017; Zeba et al., 2022). The included studies used snowball sampling ( Nath & Agarwal, 2022), systematic sampling ( Shashikala & Suresh, 2018), random sampling ( Dutta et al., 2014; Upreti & Sherpa, 2018), and stratified random sampling ( Tahir et al., 2022) as well. There was considerable variation in the sample size of the studies with sample size ranging from 75 in one study to 693 in another study.

**3.2.6 Theories, Research instrument and data collection**

There are several theories that have been used in the included studies. The Stimulus-Organism-Response (S-O-R) model was used in four studies ( Lim et al., 2022; Nath & Agarwal, 2022; Rai & Anirvinna, 2019; Shashikala & Suresh, 2018;). SERVQUAL model was used in three studies ( Biswas & Verma, 2023; Dutta et al., 2014; Shahzadi et al., 2018). One study each utilised cognitive dissonance theory ( Lim et al., 2022), DINESERV model ( Upreti & Sherpa, 2018), theory of planned behaviour ( Tahir et al., 2022) and theory of Reasoned Action ( Zeba et al., 2022).

Survey tools consisted of questionnaires and Likert scale ( Rai & Anirvinna, 2019; Tahir et al., 2022; Tinne, 2012; Upreti & Sherpa, 2018), self-administered questionnaire with close-ended questions ( Biswas & Verma, 2023; Gharpure et al., 2021; Gupta et al., 2022; Lim et al., 2022; Nath & Agarwal, 2022; Sahni & Mohsin, 2017; Shahzadi et al., 2018; Shashikala & Suresh, 2018; Zeba et al., 2022), and open-ended questionnaire ( Dutta et al., 2014). Two studies ( Biswas & Verma, 2023; Gupta et al., 2022), used location intercept method to collect responses.

**3.2.7 Data analysis methods**

The analyses used were chi-square test ( Sahni & Mohsin, 2017), gap analysis ( Shahzadi et al., 2018), factor analysis ( Biswas & Verma, 2023; Gupta et al., 2022; Lim et al., 2022; Shashikala & Suresh, 2018; Tinne, 2012), and factorial design by analysis of variance (ANOVA) ( Dutta et al., 2014; Gharpure et al., 2021; Zeba et al., 2022). Correlation analysis was used in four studies ( Gharpure et al., 2021; Rai & Anirvinna, 2019; Shahzadi et al., 2018; Upreti & Sherpa, 2018) and regression analysis were used in four studies ( Rai & Anirvinna, 2019; Shashikala & Suresh, 2018; Upreti & Sherpa, 2018; Zeba et al., 2022). Descriptive statistics (frequency and percentages) were used by Nath & Agarwal (2022) and Upreti & Sherpa (2018) in their study. Structural equation modelling was used in four studies ( Biswas & Verma, 2023; Gupta et al., 2022; Lim et al., 2022; Tahir et al., 2022).

**3.2.8 Frequency of fine dining**

Three studies reported the frequency of dining. While in one study it ranged from once a week to once a year ( Shahzadi et al., 2018), Gupta et al. (2022) reported that approximately 70% of the respondents had dined at fine dining restaurant on multiple occasions. However, the specific timeframe for these visits was not specified. Lim et al. (2022) reported that around 80% of the respondents were dining for the first time at a fine dining restaurant.

In Pakistan, the choice of food mostly varied between traditional food on one hand and Chinese or Thai food on the other ( Shahzadi et al., 2018). In Bangladesh, the preference was for local cuisine even when dining at an upscale restaurant ( Tinne, 2012).

The findings of one study indicated that while majority of the respondents preferred visiting fine dining restaurant mostly with friends and family, around 10% preferred visiting with colleagues and very few preferred to visit alone ( Shahzadi et al., 2018). This is consistent with results obtained by Lim et al. (2022). In an Indian study, dining companions were mostly friends followed by family.

**3.2.9 Outcomes**

The selected studies examined a variety of outcomes. In four studies ( Nath & Agarwal, 2022; Sahni & Mohsin, 2017; Shahzadi et al., 2018; Tinne, 2012), factors influencing choice of fine dine restaurants were studied. Customer patronage and willingness to pay were examined by Dutta et al. (2014) and Gupta et al. (2022). Customer satisfaction was investigated in most of the studies ( Biswas & Verma, 2023; Gharpure et al., 2021; Lim et al., 2022; Nath & Agarwal, 2022; Rai & Anirvinna, 2019; Shahzadi et al., 2018; Shashikala & Suresh, 2018; Upreti & Sherpa, 2018; Zeba et al., 2022). Behavioural intentions were measured in two studies ( Gharpure et al., 2021; Shahzadi et al., 2018) and customer loyalty towards fine dine restaurants was examined in two studies ( Rai & Anirvinna, 2019; Zeba et al., 2022). Lim et al. (2022) examined the connection between customer satisfaction and the word-of-mouth endorsement. Sustainable behaviour of restaurants and wastage reduction practices were investigated by one study ( Tahir et al., 2022).

***Factors affecting selection of fine dining restaurants***

In the Bangladeshi study a comprehensive assessment was conducted, and fifteen factors were studied that impact customers’ choices when considering dining at upscale establishments. The study’s results indicated that the choice of upscale restaurants is primarily influenced by the promotional aspects, restaurants’ internal attributes, situational circumstances, pricing considerations, restaurant’s image, and the overall deluxe dining experience ( Tinne, 2012). The same study indicated that many upscale restaurants go beyond merely offering exceptional cuisine; they also offer supplementary services such as dedicated children’s areas, celebrity appearances, and live performances to attract and engage consumers ( Tinne, 2012).

In the study from Pakistan, Shahzadi et al. (2018) suggested the primary factors that influenced the choice of restaurant were: taste of the food, its safety, freshness, environmental cleanliness, and pricing. Among these, taste of food stood out as the most important factor when selecting a fine dining establishment, whereas music was accorded the least significance.

Sahni & Mohsin (2017) identified service quality as the only statistically significant factor that influences the choice of fine dine restaurants. Furthermore, their study indicates that across religions, the variety of food offered by the restaurant played a role in influencing the choice of fine dining establishments.

Nath & Agarwal (2022) found that servicescape ranks as the third most influential factor—after primary product offering and price consideration—when selecting a restaurant.

***Customer satisfaction and associated factors***

Four studies examined the impact of three quality attributes: food, service, and atmospherics ( Dutta et al., 2014; Gharpure et al., 2021; Sahni & Mohsin, 2017; Shahzadi et al., 2018). In their study Gharpure et al. (2021) found overall customer satisfaction was significantly correlated with service quality—0.672 ** for helpfulness and politeness of staff, .669** for timeliness of service, and .531** for adequacy of staff and their grooming—and ambience— .545** for comfortable and welcoming feeling, .491** for availability and cleanliness of washrooms, and.516** upscale restaurant reputation. This was higher than correlation between overall customer satisfaction and food and beverage quality attributes such as portion size (.373**) and availability of the alcoholic beverages (.284**).

The same study indicated that among food quality attributes (portion size, temperature and taste, availability of alcoholic beverages and menu variety), highest correlation was found to be with temperature and taste of the food (.584**) and menu variety (.566**). The findings of two studies ( Biswas & Verma, 2023; Upreti & Sherpa, 2018) indicate that satisfaction of diners in a fine dining establishment is notably influenced by various elements of dining services including tangibility, reliability, assurance, and empathy. Upreti & Sherpa (2018) report a robust and positive correlation, ranging from 0.761 (tangibility) to 0.892 reliability), with diner satisfaction. However, Biswas & Verma (2023) reported that the food quality (β = 0.448, p< .01) as the most crucial factor influencing customer satisfaction following closely, tangibility (β = 0.328, p< .01) was ranked second, succeeded by assurance, reliability, restaurant image, and responsiveness.

The results of another study—conducted during COVID-19 pandemic—indicate that customers give importance to factors such as food quality (including sensory experience, satiety value, and menu selection), hygiene, and pricing with the fine dining experience amid the pandemic ( Lim et al., 2022). The authors present evidence to suggest that customer satisfaction mediated the relationship between various factors and word-of-mouth recommendation.

Zeba et al. (2022) report that each of the six attributes of perceived value—efficiency, service quality, social experience, enjoyment, aesthetics, and altruism—demonstrated a significant correlation with both customer delight and customer satisfaction. Additionally, the results suggest that in terms of ensuring customer satisfaction, utilitarian values exhibited higher regression coefficients than hedonic values. Conversely, when considering customer delight, the situation was reversed. When compared to customer satisfaction, customer delight exerts a more robust impact on customer loyalty and serves as a more accurate predictor of the same ( Zeba et al., 2022).

***Behavioural intentions and customer satisfaction***

Association between behavioural intentions and customer satisfaction was studied in two studies ( Gharpure et al., 2021; Shahzadi et al., 2018). Behavioural intentions such as recommending to others (r= .605, p <. 001) and being a frequent visitor (r= .608, p <. 001) were found to be positively correlated with overall satisfaction post visiting the fine dining restaurant ( Gharpure et al., 2021). However, the other study ( Shahzadi et al., 2018) reported that the relationship between restaurant attributes (food, service and atmospherics) and behavioural intentions was moderated by customer satisfaction.

***Servicescape and customer’s loyalty***

Servicescape and its relationship with different aspects of fine dining restaurants was examined in three studies ( Nath & Agarwal, 2022; Rai & Anirvinna, 2019; Shashikala & Suresh, 2018). Correlation (0.769) was found between servicescape and customers’ loyalty towards dining at a fine dine restaurant ( Rai & Anirvinna, 2019). The authors estimate that 59% of the variation in customer loyalty could be attributed to servicescape.

It was found that fine dining establishments experience higher levels of food wastage. This was attributed to the formal ambiance and frequent change of dinnerware. Additionally, the wastage has an impact on the sustainable dining practices and loyalty of patrons towards the foodservice business ( Tahir et al., 2022).

***Willingness to pay***

Dutta et al. (2014) reported that for fine dining restaurants, willingness to pay increases with a corresponding increase in quality attributes (food, service, and atmospherics). This is supported by the findings of the study by Nath & Agarwal (2022) that indicated consumers were inclined to pay more for a pleasant dining atmosphere and forego the comfort of food delivered at home. Gupta et al. (2022) report that in contrast to other factors, higher correlation was observed between a strong interest in gastronomy and the willingness to pay a higher price for the experience of luxury while dining at fine dining restaurants.

### 3.3 Quality assessment of the included studies

Out of fourteen studies, six had scores between 30-36 ( Dutta et al., 2014; Biswas & Verma, 2023; Tahir et al., 2022; Gupta et al., 2022; Lim et al., 2022; Zeba et al., 2022), four had scores between 24-29 ( Tinne, 2012; Sahni & Mohsin, 2017; Shashikala & Suresh, 2018; Shahzadi et al., 2018) and four studies had scores less than 24 ( Upreti & Sherpa, 2018; Rai & Anirvinna, 2019; Gharpure et al., 2021; Nath & Agarwal, 2022). Ethics and bias were reported in very few studies. Most of the studies scored between fair to good on items with respect to abstract, introduction and methods. Studies scoring less than 24 are generally regarded as poor to very poor on items such as transferability or generalisability, implications and usefulness, ethics and bias. The result of this quality assessment is presented in table 2.

**Table 2.** Qualitative assessment of the included studies.

Study ID	Abstract and title	Introduction and aims	Method and data	Sampling	Data analysis	Ethics and bias	Results	Transferability or generalisability	Implications and usefulness	Total Score
Tinne 2012	4	3	3	3	3	-	3	2	2	24
Dutta 2014	4	4	4	3	4	2	4	3	3	31
Sahni 2017	3	3	3	3	3	2	3	2	2	24
Shahzadi 2018	4	4	3	3	3	-	4	3	4	28
Shashikala 2018	4	3	3	3	3	-	3	3	2	24
Upreti and Sherpa 2018	4	3	3	3	2	-	3	1	1	20
Rai 2019	3	3	2	2	2	-	2	1	1	16
Gharpure 2021	4	2	3	3	3		2	1	1	19
Biswas and Verma 2023	4	4	4	3	4	3	3	3	3	31
Tahir 2022	4	4	4	4	4	-	3	3	4	30
Nath 2022	2	3	2	2	2		2	2	1	16
Gupta 2022	4	4	4	4	4	2	4	4	3	33
Lim 2022	4	4	4	4	4	3	4	4	3	34
Zeba 2022	3	4	4	4	4	3	4	4	4	34

## 4. Discussion

The objectives of this systematic review were to find out the extent of literature available on fine dining and examine factors affecting selection of fine dining restaurants—all in the context of South Asian countries. Despite the comprehensiveness of the search, the results were meagre: only fourteen articles qualified for inclusion. Out of 114 studies that underwent full text screening, majority were rejected as they were not conducted in South Asian countries.

Fine dining establishments are characterized by offering comprehensive table service along with a wide and sophisticated array of food and beverages. Such restaurants frequently provide atmosphere that is theme-based, premium tableware, well-trained, and formally attired staff ( Lee, 2021). The studies that were included in the review did not operationalize the concept of the fine dining under the “methods” section but rather introduced and described it in the “introduction” section. Furthermore, only one study indicated the average price of a meal either with or without alcohol ( Shashikala & Suresh, 2018). For fine dining or upscale restaurants, price is the primary factor that differentiates such establishments from other restaurants or establishments. These findings suggest that the authors conducting the primary search should define or operationalize the concept of fine dining, upscale restaurants, or full-service restaurants in methods section as otherwise these concepts may be used interchangeably during study.

In terms of methods, majority of the studies relied on survey method to collect quantitative data. This highlights a significant gap in the utilization of other research methods, particularly qualitative and phenomenological approaches like in-depth interviews and focus group discussions. Such non-quantitative methods are apt for uncovering nuanced contextual differences in perceptions and attitudes and some researchers have applied them in the context of fine dining restaurants ( Batat, 2020; Filimonau et al., 2020).

The findings from the included studies in this systematic review broadly indicate service quality, food quality, and atmosphere or ambience to have a significant impact on deciding the dining destination. This aligns with existing literature on factors influencing customers’ choices in fine dining restaurants ( Garg & Amelia, 2016; Mhlanga & Tichaawa, 2016). Nevertheless, augmenting the experience with supplementary offerings such as a designated children's area, celebrity appearances for promotional activities, and live entertainment may prove advantageous in luring the customers ( Tinne, 2012). It was observed that none of the studies the influence of celebrity endorsement in attracting customers despite there being some evidence indicating its importance ( Wachyuni & Priyambodo, 2020).

Considering that hygiene and health precautions have gained significant prominence during and after the pandemic, one study investigated customers’ intentions towards fine dining restaurants during the pandemic ( Lim et al., 2022). It is imperative to thoroughly examine and delve into the connection between the intention to visit upscale restaurants and cleanliness, and overall post-pandemic behaviour of the consumers while dining out at a fine dining restaurant.

The willingness to pay more (WTP) for fine dining restaurants is another aspect that was reported to substantially influence customers’ decision ( Ali & Nath, 2013; Dutta et al., 2014; Nath & Agarwal, 2022). The high cost of dining at fine dining establishments results in lower rates of return visits compared to other restaurant types ( Herrera & Young, 2023). Some studies ( Chua et al., 2020; Gupta, 2019) conducted in Asian settings indicate that for Asians consumers, menu price or average price per customer is amongst the top considerations while selecting dining-out destination across different restaurant styles (full-service, quick casual and quick service restaurants). Socio-economic status directly influences the willingness to pay (WTP) especially for individuals from weaker financial backgrounds ( Nishinakagawa et al., 2023). Unsurprisingly, for such individuals price takes precedence over quality and health aspects of food.

It is well-established that fine dining establishments often place a strong emphasis on creating a unique and memorable dining experience by manipulating elements of servicescape along with service quality. In their study, Rai & Anirvinna (2019) examined the influence of servicescape on customer loyalty in the context of upscale dining establishment.

Growing environmental consciousness has led some diners to consider a foodservice operation’s sustainability practices, including sourcing of ingredients, waste reduction, and eco-friendly initiatives. Batat (2020) reported a higher WTP among young consumers for sustainable sourcing and environment-friendly practices. This is similar to the study conducted by Tahir et al. (2022) in which the authors reported the concern of food wastage at fine dining restaurants. In fine dining restaurants, owing to the frequent change of dinnerware and the extensive menu—served in courses—the amount of food wastage is high ( Batat, 2020; Filimonau et al., 2023). Furthermore, as many fine dining restaurants offer specialised cuisine, exotic ingredients are often required. Such ingredients if sourced through unreliable supply chains may lead to wastage as chefs may discard ingredients that do not meet their quality standards. Filimonau et al. (2023) argued that factors contributing to food waste in fine dining are the visual presentation of the food and suggested streamlining policies and procedures at fine dining restaurants will help in promoting the practices to reduce food waste.

The proliferation of social media platforms has amplified the visibility and enhanced the reach of foodservice establishments. Customers increasingly rely on platforms like Tripadvisor and Facebook to make informed decisions and fine dining establishments are swiftly embracing these platforms to connect with prospective patrons ( Kung’u et al., 2022). We found that some attributes—such as use of social media—identified by other researchers as being a significant influence on the selection of fine dining restaurants, were not examined in any of the included studies.

### Strengths and limitations

The review adhered to the PRISMA reporting guideline, ensuring a methodologically robust approach. The use of transparent methods and a structured, systematic approach further enhances the credibility of this review. The studies that were included, provide a valuable insight into the factors influencing customers’ choices and behaviours, customer satisfaction and willingness to pay in a fine dine restaurants; limited to the region of South Asia. The review aimed at synthesising the literature studies employing a systematic review methodology, offering a diverse perspective on the extent of subject matter in one of the most populated regions in the world.

The authors were able to delineate several points for future research. First, the limited number of studies meeting the eligibility criteria (fourteen), indicate a potential gap in literature on this topic. This indicates limited evidence: fourteen studies from four South Asian countries—India, Pakistan, Nepal and Bangladesh. No studies from Afghanistan, Bhutan, Maldives, or Sri Lanka met the inclusion criteria. This scarcity of studies limits the generalizability of the findings. Secondly, the studies incorporated in the analysis shared a commonality in focusing predominantly on quantitative surveys, with a notable absence of qualitative studies. This limitation posed a challenge in obtaining a varied perspective. Also, the lack of an operational definition of "fine dining" in studies may introduce ambiguity and hinder the synthesis of results.

## 5. Conclusion

This systematic review focuses scientific research on different aspects of fine dining and the factors that affect the choice of fine dining restaurants in South Asia. It highlights the need for further research in the South Asian hospitality industry regarding fine dining. The review encourages discussions about diversifying research methodologies to explore consumer preferences, market trends, and the influence of economic and cultural shifts to not only meet customer expectations but also shape the future of fine dining and contribute to its continued growth and evolution.

Overall, the relationship between service quality and customer satisfaction is well-established in the literature and is a critical consideration for organizations aiming to build strong customer relationships and achieve sustainable success in the service industry. Businesses that prioritize service quality and consistently meet or exceed customer expectations are more likely to enjoy higher levels of customer satisfaction and loyalty. Continuous improvement efforts are essential to maintain and enhance customer satisfaction over time.

In conclusion, this systematic review provides a valuable overview of existing research on fine dine restaurants. Despite the importance of restaurant choice criteria and a growth in hospitality sector, there are limited published empirical studies on different aspects of fine dine in South Asian context. This paucity underscores the need for more comprehensive and standardized research in this area to establish a clearer understanding of the factors influencing customer choices and behaviours in fine dine settings. Additionally, future studies should aim to bridge the highlighted gaps literature and employ rigorous research designs to enhance the reliability and applicability of their findings.

## Ethics approval

Not applicable.

## Informed consent statement

Not applicable.

## Author contributions

Conceptualization, PD, VSD, SN. SKP; methodology PD, VSD, SN and SKP; software, PD and VSD; validation, PD, VSD, SN, KN and SKP; formal analysis, PD, and SKP; data curation, PD, VSD, and KN; writing—PD and SKP; writing—review and editing, VSD, SN, KN; visualization, PD and SKP; supervision, SKP; project administration, PD. All authors have read and agreed to the final version of the manuscript.

## Data Availability

No data are associated with this article. Figshare: Understanding Fine Dining Restaurant Dynamics in South Asia: A systematic review. https://doi.org/10.6084/m9.figshare.28350806.v1 ( Dabral, Paritosh 2025a). The project contains the following extended data:
1. Supplementary file 1 Search strategy 2. Supplementary file 2 PRISMA checklist Figshare: PRISMA flowchart. https://doi.org/10.6084/m9.figshare.28360514.v1 ( Dabral, Paritosh 2025b). The project contains the following extended data:
1. PRISMA flowchart.docx Data are available under the terms of the Creative Commons Attribution 4.0 International license (CC-BY 4.0) PRISMA Checklist for Understanding Fine Dining Restaurant Dynamics in South Asia: A systematic review. https://doi.org/10.6084/m9.figshare.28350806.v1 ( Dabral, Paritosh 2025a). PRISMA Flowchart for Understanding Fine Dining Restaurant Dynamics in South Asia: A systematic review. https://doi.org/10.6084/m9.figshare.28360514.v1 ( Dabral, Paritosh 2025b). Data are available under the terms of the Creative Commons Attribution 4.0 International license (CC-BY 4.0)
