# Supplementary material for: Understanding fine dining restaurant dynamics in south Asia: A systematic review
Source: F1000Res. 2025 Mar 10;14:282. [Version 1] doi: 10.12688/f1000research.161879.1 (PMC12033979; doi:10.12688/f1000research.161879.1)
Supplement: Peer review history

# Reviewer report: Reviewer response for version 1

**DOI:** 10.5256/f1000research.177971.r373757

**Published:** 2025-04-26

**Authors:** Ganna Demydyuk

**Affiliations:** 1 Leiden University, Leiden, The Netherlands

The manuscript is generally well-structured and follows PRISMA guidelines, enhancing its transparency. However, language and grammar should be improved throughout.

 My main reservations are about the contribution to knowledge of this study. The manuscript lacks a solid theoretical motivation for conducting a region-specific SLR. It is not clear why South Asia requires a separate review when a global synthesis of fine dining research may be more appropriate as a first step. The authors may consider first reviewing the extant literature on fine dining globally, highlighting prevailing theories, success factors, and conceptual frameworks. From there, they can argue what is different or unique about the South Asian context, and thereby justify a focused regional synthesis.

 In its current form, the manuscript does not provide this conceptual grounding or differentiation. The absence of a robust theoretical or contextual rationale weakens the case for the review. I strongly recommend that the introduction be revised to include a review of global fine dining literature and a critical argument explaining why a South Asia-specific focus is needed. 

 Furthermore, transparency is lacking in the description of the search strategy. After including the exact search strings, filters, and inclusion/exclusion criteria, authors should justify them based on the extant literature. This would allow replication, help readers understand why only 10 studies were eligible, and add strength to the theoretical contribution and positioning.

 Moreover, the sample analyzed is quite small, raising questions about the possible depth of analysis. The narrative synthesis approach is appropriate, but it remains somewhat superficial. A more structured thematic or content analysis could have uncovered deeper insights, especially given the small number of studies. Furthermore, the authors could have used basic coding or thematic mapping to strengthen the synthesis. Even simple frequency or co-occurrence tables of themes across studies would have added structure and clarity.

 Finally, the conclusions are consistent with the findings, but they remain descriptive. The implications for practitioners and researchers could be further developed. 

 Overall, the study addresses an interesting topic and has solid potential for publication; however, the improvements discussed above and summarized below are necessary to bring it to a publishable standard. 

 1. Improve grammar throughout the manuscript.

 2. Provide a strong theoretical justification for conducting a South Asia-focused review.

 3. Begin with a broader synthesis of global fine dining literature to contextualize the need for a regional focus.

 4. Acknowledge and discuss the limitations of the small sample size more explicitly and in connection with 2 and 3. Enhance methodological transparency with detailed search strings and criteria.

 4. Consider using a more structured synthesis method (e.g., thematic analysis).

 5. Deepen the discussion and practical implications of findings.

 Good luck with your revision!

Are the rationale for, and objectives of, the Systematic Review clearly stated?

Partly

Is the statistical analysis and its interpretation appropriate?

Yes

If this is a Living Systematic Review, is the ‘living’ method appropriate and is the search schedule clearly defined and justified? (‘Living Systematic Review’ or a variation of this term should be included in the title.)

No

Are sufficient details of the methods and analysis provided to allow replication by others?

Yes

Are the conclusions drawn adequately supported by the results presented in the review?

Partly

Reviewer Expertise:

Customer-centric profitability analytics, experience-based financial management, management of multiple dining venues in all-inclusive F&B settings, thresholds of customer satisfaction for profitability and investment decisions

I confirm that I have read this submission and believe that I have an appropriate level of expertise to confirm that it is of an acceptable scientific standard, however I have significant reservations, as outlined above.

# Reviewer report: Reviewer response for version 1

**DOI:** 10.5256/f1000research.177971.r373759

**Published:** 2025-04-10

**Authors:** Amit Kumar Agarwal

**Affiliations:** 1 Department of Management and Humanities, Dr Shyama Prasad Mukherjee International Institute of Information Technology, Naya Raipur, Chhattisgarh, India

I find the present article interesting and parsimonious. The authors have clearly stated the objective of identifying the factors affecting fine dining selection in South Asia. The authors also provided adequate justification for conducting the present literature review when two LRs existed. Still, none investigated South Asia, and one of the LRs only examined food service selection factors.  Hence, the need for the study is apt and in line with the scope of the journal and extant literature.

 PRISMA Methodology has been used to filter and select the articles, which is a standard methodology, and proper steps were taken to screen duplicate and irrelevant articles. Further, any other researcher can replicate the study.

 The authors used thematic analysis to segregate the filtered key studies, and dominant themes were examined to find gaps and future research areas. The study aptly concludes that Despite the importance of restaurant choice criteria and growth in the hospitality sector, there are limited published empirical studies on fine dining in South Asia.

 Based on rigour and deep analysis, I would recommend the study for acceptance in the journal.

Are the rationale for, and objectives of, the Systematic Review clearly stated?

Yes

Is the statistical analysis and its interpretation appropriate?

Yes

If this is a Living Systematic Review, is the ‘living’ method appropriate and is the search schedule clearly defined and justified? (‘Living Systematic Review’ or a variation of this term should be included in the title.)

Yes

Are sufficient details of the methods and analysis provided to allow replication by others?

Yes

Are the conclusions drawn adequately supported by the results presented in the review?

Yes

Reviewer Expertise:

Marketing, Consumer behaviour, Tourism & Hospitality, Co-creation

I confirm that I have read this submission and believe that I have an appropriate level of expertise to confirm that it is of an acceptable scientific standard.

# Reviewer report: Reviewer response for version 1

**DOI:** 10.5256/f1000research.177971.r373760

**Published:** 2025-04-09

**Authors:** Ni Made Ary Widiastini

**Affiliations:** 1 Universitas Pendidikan Ganesha, Buleleng, Indonesia

(1. Introduction)

 The author needs to provide the urgency of the research “Understanding the dynamics of fine dining restaurants in South Asia: A systematic review”. Clearly state the objectives or aims of your research.

 (2.2 Review Questions)

- It is necessary to provide information on recent evidence (years of significant development, for example the last 3 or 5 years) regarding the dynamics of fine dining restaurants and gaps in the existing literature on related aspects of fine dining restaurants in South Asia. Provide a review of the method (how the data was searched and analyzed), the results and the discussion.
- It is also need to provide an overview of aspects and factors that influence the decision to choose a fine dining restaurant, explain the methods (how data was sourced and analyzed), results, and discussion

Are the rationale for, and objectives of, the Systematic Review clearly stated?

Yes

Is the statistical analysis and its interpretation appropriate?

Yes

If this is a Living Systematic Review, is the ‘living’ method appropriate and is the search schedule clearly defined and justified? (‘Living Systematic Review’ or a variation of this term should be included in the title.)

Yes

Are sufficient details of the methods and analysis provided to allow replication by others?

Yes

Are the conclusions drawn adequately supported by the results presented in the review?

Yes

Reviewer Expertise:

tourism

I confirm that I have read this submission and believe that I have an appropriate level of expertise to confirm that it is of an acceptable scientific standard, however I have significant reservations, as outlined above.
